# Supplementary material for: WordCommentsAnalyzer: A windows software tool for qualitative research
Source: F1000Res. 2018 Sep 4;7:536. Originally published 2018 May 3. [Version 2] doi: 10.12688/f1000research.14819.2 (PMC6137411; doi:10.12688/f1000research.14819.2)
Supplement: Supplementary file 3 [file f1000research-7-17614-s0002.tgz › b2a79496-4ada-47c4-9682-a99ce867836d.zip!/Eight Iranian Health Journals/The Journal of Toloo-e-Behdasht_26.docx]

© The Journal of Toloo-e-Behdasht. This work is licensed under CC BY 4.0 (https://creativecommons.org/licenses/by/4.0/).

Volume & Issue: دوره 13 - شماره 1

Title: بررسی کارایی توده زیستی ساکارومایسس سرویسیه (مخمر نان) غیر فعال در حذف آرسنیک از محلولهای آبی

http://tbj.ssu.ac.ir/article-1-1589-fa.html

مقدمه: آرسنیک یکی از عناصر سمی متداول است که عمدتاً توسط آب در محیط انتقال می یابد. حضور آرسنیک در آب آشامیدنی از نگرانیهای اصلی بسیاری از تامین کنندگان آب در جهان است. بنابر این در این مطالعه جذب زیستی آرسنیک توسط ساکارومایسس سرویسیه (مخمر نان) غیرفعال به عنوان یک جاذب زیستی ارزان قیمت از محیط آبی انتخاب گردید. روش بررسی: این بررسی یک مطالعه تجربی در مقیاس آزمایشگاهی بوده که برروی 243 نمونه سینتیک بر اساس روش های آماری در یک سیستم ناپیوسته انجام گردید. در این مطالعه اثر فاکتورهای زمان تماس (5، 15، 30 ،60 ،120 دقیقه و 24 ساعت)، pH (5، 7 و 9)، غلظت آرسنیک( 100، 250، 500، 750 و 1000 میکروگرم در لیتر) و دوزهای جاذب (5/0، 1، 5/2 و 5 گرم در لیتر) بررسی شد. در نهایت، سینتیک جذب و ایزوترمهای تعادلی ماده جاذب مورد بررسی قرار گرفت. یافته ها: ساکارومایسس سرویسیه غیر فعال در pH برابر با 5، مقدار جاذب زیستی g/L 1 و غلظت اولیه فلز µg/L 100 دارای 49/89 % حذف آرسنیک است. ماکزیمم جذب پس از مدت زمان 60 دقیقه مشاهده نتیجه گیری: نتایج حاصل از تحقیق نشان داد که با افزایش pH محلول کارآیی جذب آرسنیک کاهش می یابد و pH بهینه در محدوده 5 می باشد. کارایی حذف آرسنیک با افزایش جرم ساکارومایسس سرویسیه تا محدوده 1 گرم در لیتر افزایش و اما با افزایش غلظت اولیه آرسنیک کاهش می یابد . حداکثر جذب در همان 15 دقیقه اول واکنش مشاهده گردید

Title: بررسی پرتوهای گاما در سنگ‌های ساختمانی موجود در شهر کرمان، سال 1392

http://tbj.ssu.ac.ir/article-1-1604-fa.html

چکیده مقدمه: برخی سنگ های ساختمانی عناصر رادیواکتیوی نظیر اورانیوم و توریوم دارند. از آنجا که انسان بطور متوسط 80 درصد وقت خود را در داخل ساختمان ها سپری می کند احتمال پرتوگیری آن توسط انسان بالا می رود. هدف از انجام این مطالعه، بررسی دزیمتری سنگ های ساختمانی در سنگفروشی های شهر کرمان در سال 1392 به منظور مشخص کردن میزان پرتوزایی آنها می باشد. روش بررسی: با مراجعه به سنگفروشی های شهر کرمان، سنگ های ساختمانی موجود و مصرفی، شناسایی و فهرستی از آنها تهیه گردید. جمعاً 275 نمونه از انواع مختلف سنگ توسط دزیمتر قلمی مدل MKS در دو حالت تعیین آهنگ دز گاما EDR))Equivalent Dose Rate برحسب میکروسیورت بر ساعت و تعیین دز معادل گاما ED (Equivalent Dose) برحسب میلی سیورت اندازه گیری شد. یافته ها: بیشترین میزان EDR در سنگ های گرانیت مورد آزمایش مربوط به گرانیت وارداتی قرمز چین به میزان 22/0، در سنگهای مرمریت، صورتی انارک به میزان 077/0، در سنگ های آهکی، زرد ماکو به میزان 03/0 و در سنگ های چینی، کرم عباس آباد به میزان039/0 میکروسیورت بر ساعت مشاهده شد. میزان ED تمامی نمونه ها برابر صفر تعیین گردید. نتیجه گیری: اگرچه میزان پرتوی اکثریت سنگ های مورد آزمایش کمتر از حد مجاز می باشد اما میزان دز سنگ های گرانیت کمی بیش از حد مجاز می باشد، از این رو توصیه می شود در مواردی که سنگ های مشابه با دز کمتر در کشور وجود دارد از ورود آنها جلوگیری شود. ضمناً تدوین استانداردهای ملی برای پرتوزایی انواع سنگ ها به منظور استفاده در ساختمان ها توصیه می شود. واژه های کلیدی: دزیمتری، سنگ های ساختمانی، کرمان، گاما

Title: ارزیابی وضعیت لاشه از نظر کلی فرم، سالمونلا و باکتریهای سرمادوست در خط تخلیه شکم و چیلر کشتارگاههای صنعتی مرغ استان یزد

http://tbj.ssu.ac.ir/article-1-1591-fa.html

مقدمه: در صورت آلودگی حتی تعداد کمی از لاشه ها به برخی از باکتری های بیماریزای غذایی، احتمال پخش این آلودگی در سردکن های آبی(چیلرها) در کشتارگاه طیور و در نتیجه، آلودگی سایر لاشه ها وجود دارد در این تحقیق وضعیت کلی فرم، سالمونلا و باکتریهای سرمادوست در دو ناحیه از کشتارگاه صنعتی مرغ در استان یزد بررسی شد. روش بررسی: نمونه گیری برای هر یک از خطوط ( کشتار و چیلر) در دو مرحله قبل از ورود و بعد از خروج از خط و در سه زمان ابتدا، وسط و انتهای زمان کشتار صورت پذیرفت. 5 لاشه مرغ بصورت کاملاً تصادفی انتخاب و شماره گذاری شده نمونه گیری از آنها در دو مرحله قبل از ورود به خط و بعد از خروج از خط با روش شستشوی کامل لاشه با استفاده از آب پپتونه صورت گرفت. در مجموع 120 نمونه اخذ شده برای کشت و شمارش به آزمایشگاه ارسال شد. یافته ها: نتایج حاکی از اختلاف معنی دار میان میزبان کلی‌فرمهای خط کشتار و چیلر بوده به عبارت دیگر میزان جمعیت کلی فرم در خط کشتار به صورت معنی داری از چیلر بیشتر بود (01/0P<). در خصوص سالمونلا تنها اختلاف معنی داری بین دو کشتارگاه مشاهده شد به طوری که در کشتارگاه یک این مقدار به طور معنی داری از کشتارگاه دو کمتر بود(5/0P<). نتیجه گیری: نتایج این تحقیق اختلاف معنی داری را بین تیمارهای مختلف در رابطه با باکتریهای سرمادوست نشان نداد.در مجموع می توان گفت که آلودگی های ثانویه در خط کشتار و افزایش تدریجی دمای آب چیلر می تواند در افزایش بارمیکروبی کلی فرم و سالمونلا موثر باشد ،اما با توجه به دمای لاشه تاثیری بر باکتریهای سرمادوست ندارد. از ا ین رو با توجه به بعضی تفاوتها بین کشتارگاه های مورد مطالعه می توان با اصلاح مدیریتی و همچنین نصب تجهیزات و امکانات مناسب در خط تخلیه شکم نسبت به کاهش بار میکروبی ناشی از آلودگی ثانویه به کلی فرم و سالمونلا اقدام نمود.

Title: برآورد متوسط زمان انتظار برای دریافت خدمت در بخش سرپایی: مطالعه موردی در درمانگاه شهید رهنمون و افشار شهر یزد

http://tbj.ssu.ac.ir/article-1-1592-fa.html

چکیده مقدمه: زمان انتظار بیان گر میزان دسترسی به خدمات و یکی از عوامل مهم است در مدیریت و سازماندهی بخش بهداشت و درمان است. زمان انتظار بیمار برای دریافت خدمات مورد نیاز همچنین یکی از ابزارهای تعیین اثر بخشی درما نگاه سرپایی می باشد. هدف مطالعه حاضر تعیین متوسط زمان انتظار بیماران مراجعه کننده به درمانگاه های شهید رهنمون و افشار شهر یزد در سال 1389 می باشد. روش بررسی: پژوهش حاضراز نوع توصیفی – تحلیلی می باشد که به صورت مقطعی در سال 1389 انجام گرفته است. جامعه مورد مطالعه شامل کلیه بیماران مراجعه کننده به درمانگاه های شهید رهنمون و افشار شهر یزد بود که از بین آنها تعداد 264 نفر به صورت تصادفی طبقه ای انتخاب و مورد مطالعه قرار گرفتند.برای جمع آروی داده ها از چک لیست استفاده شد، سپس جهت تجزیه و تحلیل آنهااز نرم افزار استفاده شد. یافته ها: متوسط زمان انتظار بیماران مراجعه کننده به درمانگاه شهید رهنمون در قسمت پذیرش، صندوق و سالن انتظار به ترتیب 7 دقیقه، 8/1دقیقه و 5/63 دقیقه و در درمانگاه افشار به ترتیب 19 دقیقه، 7/1دقیقه و 38 دقیقه بود. لازم به ذکر است میان قسمت پذیرش (00/0=P)و سالن انتظار (00/0=P)اختلاف معنی دار مشاهده شد. نتیجه گیری: استفاده از سیستم نوبت دهی تلفنی در درمانگاه های مذکور، تدوین برنامه زمان کاری مشخص برای پزشکان از سوی مدیریت درمانگاه، افزایش ساعت کاری پزشکان در درمانگاه با اولویت دادن یا سهم داشتن بیماران غیر بومی برای پذیرش و افزایش تعداد پزشکان متخصص در بخش هایی که با ازدحام بیماران همراه است می تواند زمان انتظار را کاهش دهد. واژه های کلیدی: زمان انتظار، بیمار، درمانگاه

Title: تعیین عدد پراکسید در روغنهای مصرفی قنادی ها، رستورانها و اغذیه فروشی‌های شهر گرگان در سال 1390

http://tbj.ssu.ac.ir/article-1-1595-fa.html

مقدمه: سرخ کردن زیاد روغن سبب هیدرولیز، اکسیداسیون و پلیمریزاسیون روغن می گردد. این امر در بدن رادیکال های آزاد ایجاد نموده و از علل سرطان، بیماری های التهابی، آترواسکلروز، پیری و نظایر آن می باشد. این تحقیق با هدف تعیین عدد پراکسید در روغنهای مصرفی قنادی ها، رستورانها و اغذیه فروشی‌های شهر گرگان در سال 1390 انجام شد. روش بررسی: در این مطالعه توصیفی مقطعی از تمام قنادی ها، رستورانها و اغذیه فروشی های شهر گرگان (124 مورد) به روش سرشماری در ساعاتی که روغن بیشترین حرارت را دیده بود مطابق استاندارد شماره 493 مؤسسه استاندارد وتحقیقات صنعتی ایران نمونه برداری شد. پس از انتقال نمونه ها به آزمایشگاه و تعیین عدد پراکسید مطابق با استاندارد شماره 4179 نمونه ها آنالیر شده و به وسیله نرم افزار آماری SPSS و آزمون تی جفتی تجزیه و تحلیل شد. یافته‌ها: از 43 نمونه مربوط به قنادی ها، 24 نمونه (56%) قابل مصرف و 19 نمونه (44%) غیر قابل مصرف بودند(015/0p=). از مجموع 15 نمونه مربوط به رستورانها و آشپزخانه های مرکزی، 6 نمونه (40%) قابل مصرف و 9 نمونه (60%) غیر قابل مصرف بودند (021/0p=). از مجموع 66 نمونه مربوط به اغذیه فروشی ها و ساندویچی ها، 12 نمونه (18%) قابل مصرف و 54 نمونه (82%) غیر قابل مصرف بودند(000/0p=). تفاوت معنی داری بین نمونه های قابل مصرف و غیر قابل مصرف در هر سه صنف وجود داشت(000/0p=). نتیجه‌گیری: یافته ها نشان داد عدد پراکسید در روغن های مصرفی قنادیها، رستورانها و آشپزخانه های مرکزی، اغذیه فروشیها و ساندویچی های شهر گرگان بسیار بالاتر از حد مجاز است. از این رو به نظر می رسد اجرای برنامه های آموزشی در مورد تغذیه و به کارگیری روش های صحیح طبخ و سرخ کردن مواد غذایی برای کارکناان این مراکز ضروری باشد.

Title: فراوانی انواع انگلهای روده ای در مراجعین به آزمایشگاه مرکزی وآزمایشگاه بیمارستان شهید صدوقی در شهر یزد در سال 1391-1390

http://tbj.ssu.ac.ir/article-1-1593-fa.html

چکیده مقدمه: امروزه علیرغم پیشرفتهای حاصل شده در تمام علوم و به خصوص علم پزشکی وبهداشت، همچنان بیماریهای انگلی به عنوان یک معضل بزرگ بهداشتی- اقتصادی اغلب کشورها به خصوص کشورهای در حال توسعه به شمار می آید و این کشورها را با از دست دادن نیروی انسانی زیاد، مصرف هزینه های بالا و اتلاف وقت به منظور ریشه کنی و کنترل انگل ها دچار مشکل ساخته است. روش بررسی: این مطالعه توصیفی- مقطعی گذشته نگر طی یک سال (1391-1390) در شهر یزد انجام شد. روش نمونه گیری به صورت آسان و با سرشماری ازکلیه مراجعین به آزمایشگاه مرکزی و آزمایشگاه بیمارستان شهید صدوقی یزد می باشد. نمونه های مدفوع با روش مستقیم (سرم فیزیولوژی و لوگل) تهیه شده و به وسیله میکروسکوپ نوری توسط پرسنل آزمایشگاه مرکزی و آزمایشگاه بیمارستان شهید صدوقی یزد بررسی شدند. یافته ها: نتایج آزمایش به عمل آمده و گزارش شده بر روی 33096 نمونه، ارتباط معنی داری بین فراوانی آلودگی وجنس را نشان داد (001/0P < ) که17481 نفر (52%)از مراجعین، مرد و بقیه زن بودند. 380 نفر(8/60%)از افراد آلوده مرد و 244 نفر(2/39%) از آنها زن بودند. بیشترین میزان آلودگی به ترتیب مربوط به تک یاخته ژیاردیا (4/43%) و بلاستوسیستیس هومینیس (1/40%) بود. نتیجه گیری: یافته های این مطالعه نشان می دهد که فراوانی انگلهای روده ای در استان یزد به علت آب وهوای گرم و خشک و سطح بهداشت مناسب، پایین بوده و در مورد اکثر انگل ها کاهش و در موارد معدودی، افزایش غیر محسوس نشان می دهد. لازم به ذکر است، همانند دیگر نقاط کشور بیشترین شیوع آلودگی مربوط به تک یاخته ژیاردیا و بلاستوسیستیس هومینیس در سنین پایین می باشد. واژه های کلیدی: بیماری های انگلی،انگل های روده ای، تک یاخته، کرم، یزد

Title: بررسی میزان رعایت حقوق بیماران و ضرورت آن از دیدگاه بیماران بستری شده در بیمارستان شهید صدوقی یزد-1391

http://tbj.ssu.ac.ir/article-1-1603-fa.html

مقدمه: نظام سلامت کارآمد نیازمند مشارکت فعال و رابطه ی نزدیک ارائه دهندگان خدمات بهداشتی و دریافت کنندگان این خدمات می باشد. بنابر این رعایت حقوق بیماران یک جزء جدایی ناپذیر از ارائه خدمات بهداشتی درمانی می باشد. وجود و تدوین و ابلاغ منشور نمی تواند دلیلی بر اجرا و رعایت آن باشد بنابراین ارزیابی میزان رعایت آن ضروری به نظر می رسد. هدف از این مطالعه، بررسی میزان رعایت حقوق بیماران بستری در بیمارستان شهید صدوقی و ضرورت رعایت این حقوق از دیدگاه بیماران می باشد. روش بررسی: این مطالعه توصیفی مقطعی در 3 ماهه ی آذر، دی و بهمن سال 1391 بر روی 200 نفر از بیماران بستری در بیمارستان شهید صدوقی از طریق یک مصاحبه ی حضوری انجام شد. افراد مورد مطالعه به طور تصادفی انتخاب شدند. ابزار گردآوری داده ها پرسشنامه استانداری بود که بر اساس منشور حقوق بیماران تهیه شده و در مطالعات قبلی روایی و پایایی آن تایید شده است. داده ها بعد از جمع آوری با استفاده از نرم افزار 18SPSS آنالیز شد. یافته ها: در این مطالعه در مجموع میزان رعایت حقوق بیماران 2/63% و ضرورت رعایت آن 85/76% از نظر بیماران بدست آمد. بین میزان رعایت حقوق بیماران و ضرورت آن در مجموع همبستگی مثبت و معنی دار (000/0 = P و 49/0 = R) مشاهده شد. میزان رعایت حقوق بیماران و ضرورت رعایت آن با جنس و تاهل ارتباطی نداشت ولی با تحصیلات، محل سکونت و سن بیماران ارتباط معنی داری مشاهده شد به نحوی که در بیماران ساکن شهر، تحصیلات بالاتر و سن بالاتر بیشتر گزارش شد. میزان رضایتمندی بیماران از بیمارستان با میزان رعایت حقوق بیماران در کل و در تمام حیطه ها ارتباط مستقیم معنی دار(000/0 = P) داشت. نتیجه گیری: با توجه به میزان رعایت حقوق بیماران و ارتباط آن با رضایتمندی بیماران از بیمارستان، برای افزایش رضایتمندی بیماران و بهبود ارائه خدمات به آنان رعایت منشور حقوق بیمار ضروری به نظر می رسد و یکی از راهکارهای مهم افزایش رعایت حقوق بیماران، افزایش آگاهی آنان از منشور حقوق بیمار می باشد.

Title: بررسی نتایج آزمایش اعتیاد و رابطه آن با خصوصیات مراجعین به آزمایشگاه تشخیص اعتیاد یزد

http://tbj.ssu.ac.ir/article-1-1594-fa.html

چکیده مقدمه: اعتیاد به مواد مخدر افراد سالم، مثبت و فعال جامعه را به بیمارانی مهمل و مصرف کننده تبدیل می کند. این تحقیق با هدف بررسی شیوع سوء ‌مصرف مواد اپیوئید در مراجعین به آزمایشگاه تشخیص اعتیاد یزد با تست راپید و تست کروماتوگرافی انجام شد. روش بررسی:در این تحقیق افرادی که به علل مختلف از جمله ازدواج، استخدام و جواز کسب درسالهای 86تا 88 به آزمایشگاه تشخیص اعتیاد یزد مراجعه کردند به روش توصیفی - مقطعی مورد بررسی قرار گرفتند.حجم نمونه 2790 نفر و روش نمونه گیری تصادفی بود. نخست اطلاعات دموگرافیک در پرسشنامه ثبت و سپس نمونه ادرار با حضور مامور در آزمایشگاه گرفته و با استفاده از نوار تست انیسان آزمایش راپید انجام و در صورت مثبت بودن جواب، از بقیه نمونه ادرار، آزمایش کروماتوگرافی انجام و پرسشنامه تکمیل گردید. یافته ها: در این تحقیق جمعاً 2790 نفر مورد بررسی قرار گرفتند. میانگین سنی نمونه ها 2/7 ± 9/25 سال بود. 9/62% مرد و بقیه زن بودند. 2/73% از نمونه‌ها جهت ازدواج،5/15% جهت استخدام، 3/3% جهت اخذ جواز کسب و بقیه(8%) به جهت دلایل دیگر مراجعه نموده بودند. شیوع مصرف مواد اپیوئید 3/5% با حدود اطمینان 95% از 5/4% تا 1/6% بدست آمد. بحث و نتیجه گیری: در این مطالعه چون فرد می دانسته جهت تشخیص اعتیاد مراجعه می کند و با عدم مصرف مواد در طی سه روز جواب آزمایش منفی می شود،لذا شیوع اعتیاد کم بوده و لزوم انجام آزمایش تشخیص اعتیاد بخصوص در متقاضیان ازدواج ضروری است.با محاسبه عود، مصرف مواداپیوئید در مردان 15 برابر زنان بدست آمد که معنی‌دار بود(05/0P≤ ). دیگر ریسک فاکتورهای مواد اعتیاد آور شامل سن بالاتر، پایین تر بودن سطح آموزش، شغل کارگری و آزاد، سیگاری بودن و سابقه فامیلی اعتیاد در خانواده بودند. واژه های کلیدی: آزمایش اعتیاد، میزان شیوع اعتیاد، تست راپید، یزد

Title: بررسی آگاهی، نگرش و عملکرد مردم شهرستان یزد درخصوص آنفلوآنزای خوکی (H1N1)A در سال 1389

http://tbj.ssu.ac.ir/article-1-1596-fa.html

چکیده مقدمه: آنفلوآنزا یکی از شایع ترین بیماری های عفونی است که باعث بیماری میلیون ها نفر از مردم می شود. ارزیابی دانش و نگرش عمومی در پیشگیری از انتقال شیوع بیماری بسیار ضروری می باشد چرا که تاثیر زیادی بر رفتارهای بهداشتی مردم دارد و ممکن است در کنترل بیماری امری کمک کننده باشد، لذا برآن شدیم که آگاهی، نگرش و رفتارهای مردم شهرستان یزد را در خصوص آنفلوآنزای(H1N1)A بررسی نمائیم. روش بررسی: پژوهش حاضر مطالعه ای توصیفی از نوع مقطعی می باشد که در سال 1389 بر روی 560 نفر از مردم شهر یزد انجام گرفت. روش جمع آوری اطلاعات به صورت پرسشنامه بوده که پایایی و روایی آن مورد تایید قرار گرفت.پس از وارد کردن داده ها در نرم افزار آماری SPSS با استفاده از آزمون های آماری،ANOVA ، T-test، Chi square و همبستگی پیرسون مورد تجزیه و تحلیل قرار گرفت. یافته ها: آگاهی نمونه های مورد بررسی، متوسط ولی نگرش و عملکرد آنها نسبتاً خوب بوده است. همبستگی مثبتی بین آگاهی و نگرش(019/0 =(p، آگاهی و عملکرد(001/0>P) و نگرش و عملکرد(004/0 =(p وجود داشت و زنان از عملکرد بهتری نسبت به مردان برخوردار بودند(001/0 =(p. بحث و نتیجه گیری: با توجه به نتایج این پژوهش افزایش اطلاعات مردم از طریق برنامه های مدون آموزشی، پوستر، پمفلت و همچنین از طریق رسانه های ارتباط جمعی (رادیو، تلویزیون) و پزشک پیشنهاد می گردد. واژه های کلیدی: آگاهی، نگرش، عملکرد، آنفلوآنزای خوکی(A(H1N1

Title: أثیر 12 هفته تمرین منتخب پیلاتس بر کیفیت زندگی مردان سالمند غیر ورزشکار

http://tbj.ssu.ac.ir/article-1-1597-fa.html

مقدمه: سالمندی، دورانی حساس از زندگی بشر می باشد و توجه به مسایل و نیازهای این مرحله، یک ضرورت اجتماعی است. هدف اصلی پژوهش حاضر، بررسی تأثیر 12 هفته تمرین منتخب پیلاتس بر کیفیت زندگی مردان سالمند غیر ورزشکار می باشد. روش بررسی‌: این مطالعه به روش نیمه تجربی انجام شد. 40 مرد سالمند غیر ورزشکار به روش تصادفی، از جامعه در دسترس مردان سالمند که در سال 1391 به مرکز بهداشتی و درمانی منطقه اردهال کاشان مراجعه کرده بودند؛ به عنوان نمونه انتخاب، و به صورت تصادفی، در دو گروه مساوی تجربی (سن2/3 ± 13/65، وزن91/6 ± 40/70، قد 91/7 ± 165)، و کنترل (سن 53/2± 86/64، وزن 940/4± 13/61، قد 73/4 ± 20/163) جای گرفتند. ابعاد مختلف کیفیت زندگی آزمودنی‌ها، از طریق پرسشنامه کیفیت زندگی-26 (Quality of Life Questionnaire-26)، اندازه گیری شد. سپس گروه تجربی به مدت 12 هفته برنامه ورزشی پیلاتس را اجرا کردند. طی دوره تمرین، آزمودنی‌های گروه کنترل هیچ گونه فعالیت ورزشی منظمی نداشتند. برای طبیعی بودن توزیع داده ها از آزمون کالموگروف- اسمرنوف، و برای ارزیابی اثر بخشی تمرینات از تحلیل کوواریانس و آزمون T در سطح معناداری 05/0 P≤ استفاده شد. یافته ها: اختلاف معناداری در کیفیت زندگی، ابعاد سلامت جسمانی (9/146F= ، 0001/0P= )، سلامت روانی (5/186F=، 0001/0P=)، روابط اجتماعی (6/335F=، 0001/0P=)، سلامت محیطی (5/287F=، 0001/0P=)، و کیفیت زندگی (کلی) (3/69 F=، 0001/0P=) سالمندان درگروه تجربی بدست آمد (05/0≥P). اما در گروه شاهد تفاوت معناداری مشاهده نشد (05/0P≥). نتیجه گیری: در مجموع 12 هفته تمرین پیلاتس، موجب بهبود کیفیت زندگی و متغیرهای مرتبط، در سالمندان مرد غیر ورزشکار شد. این شیوه می تواند به عنوان یک راهکار غیرتهاجمی و غیر دارویی برای ارتقای سطح زندگی سالمندان پیشنهاد شود.

Title: مقایسه میزان سازگاری اجتماعی در کودکان نابینا و عادی در مدارس ابتدایی شهر مشهد

http://tbj.ssu.ac.ir/article-1-1598-fa.html

مقدمه: اجتماعی شدن یک فرایند دو جانبه ارتباطی میان فرد و جامعه است. به همین دلیل جریانی پیچیده به شمار می آید که ابعاد و جوانب گوناگونی دارد. از جمله این ابعاد می توان به کسب مهارت ها ی اجتماعی، چگونگی برقراری ارتباط با سایر افراد و سازگاری اجتماعی اشاره کرد. محیط خانواده، مدرسه و وسایل ارتباط جمعی در فراهم ساختن امکان یادگیری سازگار با محیط نقش و مسئولیت مهمی بر عهده دارند. این بررسی با هدف مقایسه میزان سازگاری اجتماعی در کودکان عادی و نابینا ی مدارس ابتدایی شهر مشهد انجام گردید. روش بررسی: این مطالعه از نوع توصیفی، تحلیلی می باشد و تعداد 270 نفر از دانش آموزان نابینا و عادی مقطع ابتدایی شهر مشهد در سال تحصیلی92-91 شرکت داشتند. دانش آموزان نابینا به صورت سرشماری و دانش آموزان بینا به روش طبقه ای خوشه ای و تصادفی از بین مدارس عادی انتخاب گردیدند. روش جمع آوری داده ها با استفاده از پرسشنامه استاندارد سازگاری اجتماعی بود که این پرسشنامه در سال 1974 در آمریکا جهت استفاده برای دانش آموزان 7 تا 13 ساله طراحی شده است. این آزمون شامل 11 خرده مقیاس، 38 زیر مجموعه و 260 سؤال می باشد. تجزیه و تحلیل داده ها با استفاده از آمار توصیفی و استنباطی با استفاده از روش t تست برای آزمون فرضیه ها استفاده گردید(05/0 P <). یافته ها: مقایسه میانگین نمرات سازگاری اجتماعی این دانش آموزان نشان داد تفاوت معنی داری بین بینایان و نابینایان از لحاظ آماری وجود ندارد (8/0=P). نتایچ میانگین سازگاری اجتماعی بین دختران نابینا و پسران نا بینا از نظر آماری معنی دار نبود(10/= P) ولی بنابر نتایج، ناسازگاری در پسران بیشتر از دختران می باشد. در این مطالعه ناسازگاری پسرها ی بینا به مراتب بیشتر از دختران بینا بوده که از نظر آماری اختلاف معنی دار می باشد (00/0 = P). نتیجه گیری: با توجه به نتایج، نا سازگاری اجتماعی در دانش آموزان دختر نابینا بیشتر از دانش آموزان دختر بینا بود. در مجموع این ناسازگاری به مراتب در پسرها بیشتر از دختران می باشد لذا توجه به آموزش و برنامه ریزی علمی و منسجم برای آنها توصیه می گردد.

Title: مقایسه میزان افسردگی، اضطراب و استرس در زنان شاغل و خانه دار شهر شیراز در سال 1391

http://tbj.ssu.ac.ir/article-1-1599-fa.html

چکیده مقدمه: طی چند دهه اخیر، افزایش میزان اشتغال زنان در جامعه باعث شده است که آنها در معرض عوارض استرس زای متعدد محیط کار از جمله افسردگی قرار گیرند. با توجه به نقش مهم زنان در جامعه و خانواده، این مطالعه به منظور مقایسه میزان افسردگی ، اضطراب و استرس در زنان شاغل و زنان خانه دار شهر شیراز انجام شده است. روش بررسی: مطالعه حاضر به صورت توصیفی تحلیلی و به روش مقطعی بر روی زنان شاغل و خانه دار شهر شیراز با استفاده از پرسشنامه 21DASS انجام شده است. این پرسشنامه برای هر یک از حیطه های افسردگی- اضطراب- استرس 7 سوال دارد و دامنه پاسخ به سوالات اصلاً، کم، متوسط و خیلی زیاد می باشد و به هر سوال به ترتیب نمره 1 الی 4 تعلق می گیرد. روش نمونه گیری بصورت خوشه ای است. مجموعا 233 نفر انتخاب شدند که 107 نفر شاغل و 126 نفر خانه دار بودند. داده های جمع آوری شده وارد نرم افزار16SPSS و با استفاده ازآماره های توصیفی و آزمونMan Witney تجزیه و تحلیل شدند. یافته ها:در این مطالعه میانه نمره اضطراب و افسردگی به ترتیب در زنان شاغل 10و 9 و در زنان خانه دار 10و10 بدست آمد که این اختلاف از نظر آماری معنی دار نبود. (05/0

Title: بررسی کارایی فرآیند الکتروکواگولاسیون با جریان متناوب و مستقیم در حذف سرب از فاضلاب صنعت باتری سازی با استفاده از الکترودهای میله ای آهنی و فولاد ضد زنگ

http://tbj.ssu.ac.ir/article-1-1600-fa.html

مقدمه: سرب از سمی ترین فلزات سنگین غیر ضروری موجود در محیط زیست بوده که از نظر سمیت، در زمره آلاینده های درجه اول قرار می گیرند. در این مطالعه کارایی الکتروکواگولاسیون با جریان متناوب و مستقیم با استفاده ازالکترودهای میله ای آهن و فولاد ضدزنگ در حذف سرب از فاضلاب صنعت باتری سازی مورد بررسی قرار گرفته است. روش بررسی: نمونه های فاضلاب از صنعت باتری سازی گرفته شد. در سلول الکتروشیمیایی از الکترودهای میله ای آهن و فولاد ضدزنگ استفاده و اتصال آنها به یکدیگر به صورت سری و به منبع تغذیه به صورت تک قطبی صورت گرفت. یافته ها: کاربرد جریان متناوب، با الکترود آهن در دانسیته جریانmA/cm26 موجب حذف سرب تا 7/96 %شد و با الکترود فولاد ضد زنگ در دانسیته جریانmA/cm28 برابر با 8/93 % بدست آمد. با کاربرد جریان مستقیم حذف بهینه سرب با الکترود آهن به ترتیب برابر با 2/97 % در دانسیته جریانmA/cm26 و با الکترود فولاد ضدزنگ برابر با 2/93 % در دانسیته جریان mA/cm28 حاصل شد. نتیجه گیری: نتایج حاصل ازاین تحقیق با موارد مشابه انجام شده توسط محققین دیگرهمخوانی دارد. با توجه به داده های حاصل شده این فرایند می تواند به عنوان روشی مناسب برای توسعه فرایند الکتروکواگولاسیون در مقیاس صنعتی در حذف سرب از محیط های آبی مطرح گردد.

Title: اعتقادات پرسنل بهداشتی درمانی شهرستان تفت در خصوص هپاتیت ب: کاربرد الگوی اعتقاد بهداشتی

http://tbj.ssu.ac.ir/article-1-1602-fa.html

چکیده مقدمه: حدود دو و نیم میلیون نفر در ایران به هپاتیت ب، به عنوان دومین علت شایع هپاتیت حاد، مبتلا می باشند. پرسنل گروه پزشکی و افرادی که با خون و مایعات بدن بیماران سر و کار دارند بیش از جمعیت عادی در معرض خطر بوده و به بیماری مبتلا می‌شوند. لذا پیشگیری در این گروه حائز اهمیت است. یکی از مدل‌های تغییر رفتار در آموزش بهداشت، الگوی اعتقاد بهداشتی است که روی تغییر در اعتقادات تمرکز دارد. این مطالعه با هدف تعیین اعتقادات پرسنل بهداشتی درمانی شهرستان تفت در خصوص هپاتیت ب بر اساس الگوی اعتقاد بهداشتی انجام شد. روش بررسی: این مطالعه‌ توصیفی و به روش مقطعی بود که در آن ﺗﻌـﺪاد 351 ﻧﻔـﺮ از پرسنل بهداشتی درمانی شهرستان تفت به روش سرشماری، وارد ﻣﻄﺎﻟﻌﻪ ﺷﺪﻧﺪ. اطلاعات توسط پرسشنامه جمع آوری و با استفاده از نرم افزار آماری SPSS ویرایش هجدهم و با کمک آمار توصیفی و آزمون‌های تحلیلی چون ضریب همبستگی پیرسون، آنالیز واریانس یک طرفه و تی تست مستقل در سطح معناداری 05/0 مورد آزمون قرار گرفت. یافته ها: در این مطالعه میانگین سن شرکت کنندگان 13/8 ±03/35 سال بود. میانگین نمره کسب شده در هر یک از سازه‌ها عبارتند از: آگاهی 67/2±73/11 (محدوه نمره 14-0)، حساسیت درک شده 1/3±41/15 (محدوه نمره 20-4)، شدت درک شده 84/2 ±17/15 (محدوه نمره 25-5)، منافع درک شده 49/3±59/12 (محدوه نمره 15-3)، موانع درک شده6/2 ±68/10 (محدوه نمره 20-4) و راهنما برای عمل 35/1±66/2 (محدوه نمره 4-0). در این مطالعه همبستگی مستقیمی میان حساسیت درک شده و آگاهی (001/0P<، **498/0=r) وجود داشت. ارتباط آماری معناداری میان سطح تحصیلات با میانگین نمره آگاهی (001/0>P)، حساسیت درک شده (001/0>P)، منافع درک شده (02/0=P) و راهنما برای عمل (036/0=P) مشاهده شد. همچنین میان محل کار شرکت کنندگان در مطالعه و میانگین نمره آگاهی (028/0=P)، شدت درک شده (001/0=P)، منافع درک شده (001/0>P) و راهنما برای عمل (001/0>P) ارتباط آماری معناداری وجود داشت. نتیجه گیری: عقاید شرکت کنندگان در سطح قابل قبولی قرار داشت. با توجه به وجود شرایط مخاطره آمیز متعدد پیرامون این افراد، اهمیت و ضرورت آموزش مداوم در خصوص پیشگیری و مقابله با انتقال این بیماری در کارکنان بخش سلامت احساس می‌شود. از این رو، آموزش مداوم آشنایی با مسائل و نحوه برخورد با بیماران و مراقبت از آنان پیشنهاد می‌گردد. واژه‌های کلیدی: هپاتیت ب، الگوی اعتقاد بهداشتی، پرسنل بهداشتی درمانی.

Title: پیشگیری و بهبود ناراحتی های روحی ، روانی از منظر کتاب و سنت

http://tbj.ssu.ac.ir/article-1-1601-fa.html

چکیده با توجه به مشکل افسردگی که بعضی از افراد جامعه به آن مبتلا هستند و افراد مبتلا به لحاظ کم‌حوصلگی از بسیاری مزایای زندگی محروم می‌شوند، هر نوع راهکار در پیش‌گیری و رفع آن دارای اهمیت است. شیوه‌های درمان دارویی و همین‌طور مشاوره کمک مناسبی است. ولی نباید آموزه‌های دینی را در این رابطه نادیده گرفت. بعضی موارد مؤثر در این رابطه مثل خودباوری، توجه به داشته‌ها و توانمندی‌هایی که شخص از آن‌ها بهر‌ه‌مند است، استمداد از ذات مقدس الهی و دوام توجه و اعتماد به خداوند، ارزیابی صحیح از زندگی دنیا و آخرت، توجه مناسب به دیگران و پرهیز از بدخواهی آنان، پرهیز از تنهایی و بیکاری، مسافرت کردن و استفاده از تفریحات سالم و کتاب‌خوانی در این مقاله مورد توجه قرار گرفته است.
